# Anti-Interleukin 5 (IL-5) and IL-5Ra Biological Drugs: Efficacy, Safety, and Future Perspectives in Severe Eosinophilic Asthma

**DOI:** 10.3389/fmed.2017.00135

**Published:** 2017-08-31

**Authors:** Diego Bagnasco, Matteo Ferrando, Gilda Varricchi, Francesca Puggioni, Giovanni Passalacqua, Giorgio Walter Canonica

**Affiliations:** ^1^Allergy and Respiratory Diseases, DIMI Department of Internal Medicine, University of Genoa, IRCCS AOU San Martino-IST, Genoa, Italy; ^2^Department of Translational Medical Sciences, Division of Clinical Immunology and Allergy, University of Naples Federico II, Naples, Italy; ^3^Department of Internal Medicine, Respiratory Disease Clinic, IRCCS Humanitas Clinical and Research Center, Humanitas University, Milan, Italy

**Keywords:** interleukin 5, precision medicine, personalized medicine, severe asthma, monoclonal antibodies, biomarkers, eosinophils, safety

## Abstract

The definition of asthma has changed considerably in recent years, to the extent that asthma is no longer considered a single disease but a heterogeneous disorder that includes several phenotypes and, possibly, endotypes. A more detailed analysis of the immunological mechanisms underlying the pathogenesis of asthma shows interleukin 5 (IL-5) to be a crucial cytokine in several asthma phenotypes. In fact, IL-5 exerts selective action on eosinophils, which, in turn, sustain airway inflammation and worsen asthma symptoms and control. Clinical trials have shown drugs targeting IL-5 or its receptor alpha subunit (IL-5Ra) to be a promising therapeutic approach to severe asthma, whose characteristics render standard therapy of little use: systemic corticosteroids only partially control the disease and have well-known adverse effects, and omalizumab is used for allergic subtypes. Analysis of the design process of clinical trials reveals the importance of patient selection, taking into account both clinical data (e.g., exacerbations, lung function, and quality of life) and biomarkers (e.g., eosinophils, which are predictive of therapeutic response).

## Introduction

The definition of asthma has changed considerably during the last decade, to the extent that, rather than a single disease based on a reversible airway obstruction, asthma is now considered a heterogeneous disease with several phenotypes (1). The turning point was the sharp division of bronchial asthma into two large groups based on the expression of the type 2 helper T lymphocyte (T_H_2) genes underlying the disease, namely, T_H_2-high and T_H_2-low asthma (2). This distinction made it easier to evaluate the disease in terms of its pathogenic mechanisms and of the drugs administered at different levels of the inflammatory cascade. Evidence of the importance of this distinction in allergy and asthma can be seen in the fact that eosinophilic inflammation led researchers to intervene directly in pathogenic mechanisms to better control the disease and reduce the number of exacerbations. The demonstration of a close link between eosinophils and interleukin (IL) 5 shifted attention to this cytokine and led to the development of new drugs able to act directly on interleukin 5 (IL-5) and its specific receptor α-subunit (IL-5Ra).

## The T_H_2-High Phenotype: the Discovery of a Special Kind of Inflammation

The clinical evidence of heterogeneity in asthmatic patients in terms of disease severity, age at onset, allergic sensitization, response to treatments, and natural history prompted researchers to better understand the pathophysiological mechanisms underlying asthma by subdividing patients into various phenotypes (3, 4). Different approaches have been proposed for dividing the groups. The Severe Asthma Research Program (SARP) study identified four asthma clusters based on age at onset, airflow limitation, comorbidities, and lung function (5). A hierarchical cluster analysis performed in the SARP study classified asthmatic patients into five groups according to age at onset, atopy, use of corticosteroids, and lung function (6). Schatz and colleagues performed a *post hoc* analysis, “The Epidemiology and Natural History of Asthma: Outcomes and Treatment Regimens” (TENOR) study, which confirmed the existence of various clusters and phenotypes in severe asthmatic adolescents and adults (7). Furthermore, in a study based on a molecular approach, Woodruff and colleagues discovered that IL-13 can stimulate the expression of chloride channel, calcium-activated, family member 1 (CLCA1), periostin, serine peptidase inhibitor, clade B (ovalbumin), and serpin family B member 2 (serpinB2), all of which are overexpressed in asthmatic patients (8). A further step forward in the knowledge of this disease was made observing that cytokines involved in its pathogenesis were not the same in all asthmatic patients, therefore allowing to subdivide them in two different groups according to the presence, or the absence, of T_H_2 inflammation. T_H_2-high patients are characterized by the expression of IL-5 and IL-13, airway hyperresponsiveness, responsiveness to inhaled corticosteroids (ICS), high serum IgE levels, and blood and airway eosinophilia. In contrast, the T_H_2-low (healthy) group does not present these characteristics (2, 3, 9). In the T_H_2-high phenotype, which is characterized by eosinophilic inflammation, IL-5 is a central cytokine, with a key role in eosinophil differentiation, survival, activation (10, 11), and migration in the lungs (12, 13). In the T_H_2-low phenotype, on the other hand, the association between inflammation and the action of the abovementioned cytokines is less well defined, and the mechanisms underlying the disease in these patients remain little known (14, 15).

## Interleukin 5

Interleukin 5 is a 13-amino acid protein that forms a 52-kDa homodimer related to both granulocyte-macrophage colony-stimulating factor (GM-CSF) and IL-3. It binds to a heterodimer receptor on eosinophils formed by the α subunit (IL-5Ra) and the βc subunit, which is shared with the IL-3 and GM-CSF receptors (16). IL-5 is synthesized and secreted by eosinophils, T_H_2 cells, mast cells, CD34+ progenitor cells, natural killer (NK) T cells, and type 2 innate lymphoid cells (ILC2) (10, 17). In asthmatic patients, CD4 T_H_2 cells, CD34+ cells, mast cells, and eosinophils are major factors in the production of IL-5. Together with IL-3 and GM-CSF, IL-5 plays an essential role (16) in inflammation and the allergic response, favoring the production, maturation, proliferation, recruitment, differentiation, and survival of eosinophils (18, 19). In addition, IL-5 in bone marrow favors the differentiation of several CD34+ cells into eosinophils (20). Strikingly, IL-5 is associated not only with active inflammation but also with airway remodeling processes (21). Moreover, IL-5 can also affect basophil and mast cell activity, owing to the common expression of several crucial receptors (IL-5R, IL-3R, IL-4R, IL-2Ra, and GM-CSF) in these cells (22).

ILCs are characterized by their lack of T-cell and B-cell receptors (TCRs and BCRs, respectively) (23) and associated with tissue repair (24), the duration of the initial immune response to microorganisms (25), and control of proliferation of commensal microorganisms (26). These cells are able to produce cytokines quickly in response to chemical and environmental signals (i.e., IL-25, IL-33, thymic stromal lymphopoietin, and IL-1β) and can act on ILC growth and differentiation (27). ILCs can be subdivided into three different categories (ILC1s, ILC2s, and ILC3s), according to the production and expression of cytokines and transcription factors (28). Further differentiation into the different subtypes of ILCs depends on the phenotypic and functional characteristics of the T-cell subset and the expression of regulatory genes, so that ILC1s are linked to T_H_1 inflammation, ILC2s to T_H_2-induced inflammation, and ILC3 to T_H_17 and T_H_22 inflammation (29). Furthermore, the transcription of several genes, including GATA-binding protein 3 (GATA3) (30) and retinoic acid receptor-related orphan receptor-α for ILC2s (RORα) (31), is related to the differentiation of the ILC precursor in ILC2s. In lung tissue, they have a role in production of IL-5, suggesting a possible effect on the development, maturation, and action of eosinophils. The discovery that ILC2s play a role in the development and maturation of T_H_2 cells makes them interesting as a possible future therapeutic target in T_H_2-high patients (29, 32).

## Eosinophils in Asthma

Interleukin 5 acts on several types of cells. However, in airway disease in general and asthma in particular, eosinophils remain its primary target. Many papers support the pleiotropic effects of eosinophils in several asthma phenotypes (33–36). First, they play a role in the innate response to exogenous agents in airways, and second, they play a role in the modulation of the adaptive immune cascade, making them important cells in the body’s defense system. Eosinophils damage tissues by degranulating and releasing reactive oxygen species and cysteinyl leukotrienes (LT) (37). *In vivo* studies have demonstrated that exposing circulating eosinophils to IL-5 can activate their degranulation processes (38). IL-5 also stimulates eosinophilic airway inflammation and airway hyperresponsiveness (39). Eosinophil secretory granules contain not only histaminase and arylsulfatase, which are directly involved in allergic reactions, but also eosinophil peroxidase, eosinophil cationic protein, major basic protein, and eosinophil-derived neurotoxin. In addition, during degranulation, eosinophils produce IL-5 and LT such as LTC4, LTD4, and LTE4 (40), which are involved in bronchoconstriction and secretion of mucus (16, 41), thus resulting in intensification of airway narrowing.

**Figure 1:**
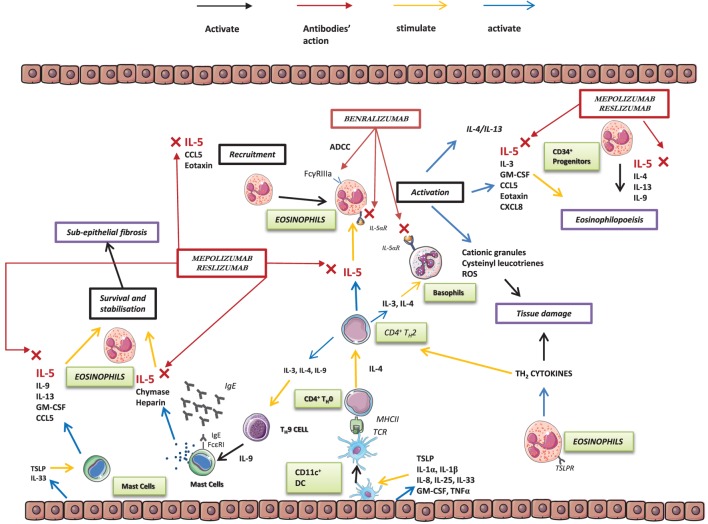
The role of interleukin 5 (IL-5) and eosinophils in airways.

Severe forms of eosinophilic asthma are characterized by intense symptoms associated with poor disease control and high eosinophil levels in blood and sputum (42). Eosinophils also play a role in the development of asthma by thickening the reticular basement membrane through production of several factors (i.e., TGF-b, VEGF, MMP-9, TIMP-1, and IL-13) (43) (Figure 1).

## From Eosinophils to Monoclonal Antibodies

Given the crucial role of IL-5 in eosinopoiesis in bone marrow and eosinophil recruitment and survival in peripheral tissues, several trials evaluated the possibility of using antibodies that target this cytokine (44, 45) or its receptor alpha subunit (46, 47) to regulate eosinophilic inflammation in uncontrolled and symptomatic asthmatic patients with high eosinophil counts.

### Mepolizumab

Mepolizumab is a humanized monoclonal *N*-glycosylated IgG1/k antibody that binds the α-chain of IL-5, thus preventing its association with the α subunit of the IL-5 receptor (48). Mepolizumab has been tested in several diseases, including severe asthma, atopic dermatitis, and nasal polyposis. Clinical trials were also conducted to assess its direct action on eosinophilic inflammation in patients with eosinophilic esophagitis, hypereosinophilic syndromes, and eosinophilic granulomatosis with polyangiitis (34, 35) (NCT02020889, NCT00266565, NCT00716651, and NCT00527566) and chronic obstructive pulmonary disease (COPD) (NCT01463644, NCT02105961, and NCT02105948) (49).

The first evaluation of the possible effect of mepolizumab was carried out in a population of cynomolgus monkeys. Hart et al. observed a significant reduction in both blood and airway eosinophilia after a single dose (50).

Since the results of mepolizumab in asthmatic patients are generally favorable, it was recently approved by the United States FDA with the trade name Nucala^®^ as add-on therapy (100 mg subcutaneously every 4 weeks) in patients aged ≥12 years with severe asthmatic eosinophilia.^1^^,^^2^ Furthermore, mepolizumab was recently approved by the European Medicines Agency Committee for Medicinal Products for Human Use.^3^

### Reslizumab

Reslizumab is an IgG4/k humanized monoclonal antibody (51) that blocks circulating IL-5 and prevents it from binding to eosinophil receptors. Several clinical trials have evaluated the use of reslizumab in asthma, hypereosinophilia after administration of diethylcarbamazine for treatment of Loa-Loa infection (NCT01111305), and eosinophilic esophagitis (NCT00635089) (52). Reslizumab was recently approved for intravenous administration in the USA in patients aged ≥18 years as add-on therapy for severe uncontrolled eosinophilic asthma (see text footnote 2).

### Benralizumab

Benralizumab differs from the abovementioned biologicals, since it targets the IL-5Ra subunit (53), which is expressed in eosinophils and basophils (54). Consequently, the effects of benralizumab were demonstrated not only for eosinophils but also for basophils. The drug induced apoptosis through antibody-dependent cell-mediated cytotoxicity (ADCC), where NK cells target cells and induce their cytotoxic action (55). The mechanism of action of benralizumab differs from that of other anti-IL-5 monoclonal antibodies, first because of its action on the receptor and second because of its higher affinity to human FcγRIIIa, resulting in enhanced ADCC action (56). In fact, through its enhanced ADCC activity, benralizumab reduces levels of circulating eosinophils and basophils (53). Therefore, it has been proposed as a biological drug not only in eosinophilic asthma but also in COPD (57), hypereosinophilic syndrome, and chronic rhinosinusitis.

### Clinical Results

Preclinical studies on monkeys, guinea pigs, and mice reported variable results for eosinophils in blood and bronchoalveolar lavage fluid, airway hyperresponsiveness, and pulmonary resistance (58) but showed a significant reduction in both blood and sputum eosinophilia in treated animals (59). Leckie et al. performed a double-blind, randomized, placebo-controlled study to test a single dose of anti-IL-5 antibody (2.5 or 10 mg/kg) in 24 non-smoking male patients with mild allergic asthma. On days 8 and 29 after administration, all patients underwent a histamine challenge, an inhaled allergen challenge, and sputum induction. The results demonstrate that infusion of anti-IL-5 antibody decreases blood eosinophil levels for up to 4 weeks and sputum levels at 4 weeks, thus leading the authors to consider this antibody a possible new approach in eosinophilic asthma (60). Further studies on this biological drug did not reveal significant variations in clinical parameters (e.g., airway hyperresponsiveness, FEV_1_, and peak flow recordings) between the mepolizumab- and placebo-treated groups. Flood-Page and colleagues treated 24 patients with 3 doses of 750 mg of mepolizumab at 0, 4, and 8 weeks and found non-significant variations in airway hyperresponsiveness, lung function (FEV_1_), the late asthmatic reaction to inhaled allergen, and peak flow recordings between placebo- and mepolizumab-treated groups, concluding that the role of eosinophils remains uncertain in asthma (61). As an explanation, the authors hypothesized that the effect of mepolizumab could not sufficiently deplete airway eosinophils and consequently act on respiratory function. Several hypotheses have been suggested by other authors. Kay and Menzies-Gow considered the possibility of different levels of depletion of eosinophils in bone marrow and airways, assuming that the antibody could not penetrate tissue or act systemically to reach bone marrow and bronchial mucosa (62). A second hypothesis allowed for the possibility that other cytokine mechanisms, such as IL-3 and GM-CSF, could overcome the mechanism blocked by mepolizumab. The authors therefore evaluated mepolizumab in more characteristic asthmatic patients to better identify subgroups of responders (63). In fact, subsequent studies were restricted to patients with severe asthma and high blood eosinophil counts and showed a significant difference in the exacerbation rate between actively treated patients and patients receiving placebo. In the first large-scale trial in this area (DREAM study), the reduction in exacerbations was significantly greater in the mepolizumab group than in the placebo group (48% for the 75-mg dose, 39% for the 250-mg dose, and 52% for the 750-mg dose) (45).

The steroid-reducing effects of mepolizumab were evaluated in the SIRIUS study, where 135 patients with severe eosinophilic asthma were treated with 100 mg of subcutaneous anti-IL-5 antibody or placebo every 4 weeks over a period of 20 weeks. After the period of optimization of the dose of oral corticosteroids (OCS), patients started to receive the drug or placebo and reduce their intake of OCS during the period from week 4 to 20. During the second part of the study (maintenance phase), no further adjustments were made to OCS doses. Eligible patients had to have undergone at least 6 months of maintenance OCS therapy (5–35 mg of prednisone or equivalent) and had an eosinophil count of ≥300/μL during the previous year and ≥150/μL during the optimization phase. The results show that patients receiving mepolizumab were 2.65 times more likely to reduce the dose of OCS than patients receiving placebo (95% CI, 1.25–4.56; *P* = 0.008), with a reduction in the frequency of exacerbations of 32% and an improvement in quality of life measured according to the Asthma Control Questionnaire 5 (ACQ-5) score (64).

In the MENSA study, mepolizumab was administered at 75 mg intravenously or 100 mg subcutaneously to 385 asthmatic patients aged between 12 and 82 years with recurrent exacerbations. In the first group, who received intravenous mepolizumab, the exacerbation rate decreased by 32%, whereas in the second group, who received mepolizumab subcutaneously, the rate fell by 53%, that is, significantly higher in patients receiving mepolizumab subcutaneously (44). In a further two major trials, the number of exacerbations was significantly reduced in patients receiving mepolizumab (45, 65). As previously described, the reduction in the frequency of exacerbations in treated patients is certainly one of the most interesting results from clinical trials. After examining the results of clinical trials with mepolizumab in severe asthmatic patients, the Cochrane Collaboration concluded that the best results for reduction in the exacerbation rate were seen in patients with elevated blood eosinophil counts. In fact, two studies, MENSA and DREAM, demonstrated that a significant clinical reduction in exacerbation rates occurred in hypereosinophilic patients (RR, 0.52; 95% CI, 0.43–0.64; participants = 690). Nevertheless, an analysis of four studies with heterogeneous serum eosinophil levels showed a non-significant difference between the mepolizumab group and the placebo group in terms of decreased frequency of exacerbations (RR, 0.67; 95% CI, 0.34–1.31; participants = 468; *I*2 = 59%) (66). Another meta-analysis of the efficacy of mepolizumab in asthmatic patients demonstrated that patients in the placebo group had more exacerbations (173 of 324; 53.4%) during the study than the mepolizumab group (91 of 310; 29.3%). The pooled analysis evidenced a significant reduction in the risk of exacerbation (0.30; 95% CI, 0.13–0.67, *P* = 0.004) (67). The most recent study on mepolizumab (MUSCA) evaluated the effect of the drug on health-related quality of life (HRQOL). This randomized, double-blind, placebo-controlled, parallel-group, multicenter, phase 3b trial included 274 patients in the mepolizumab arm and 277 in the placebo arm, both with a history of at least two exacerbations requiring treatment during the previous year, despite daily use of high-dose ICS combined with other controller medicines. A significant change was observed in the St. George’s Respiratory Questionnaire score at week 24, with an improvement in symptoms and HRQoL in patients receiving mepolizumab, compared with those receiving placebo (68).

The other IL-5 antagonist, reslizumab, also displayed encouraging results. Castro et al. reported a significant reduction in asthma exacerbation rates in a phase 3 study of patients aged 12–65 years with asthma that was inadequately controlled using medium–high doses of ICS. Blood eosinophil counts were >400/μL, and patients had experienced one or more exacerbations in the previous year (69).

The reduction in the exacerbation rate was also the primary outcome measure in clinical trials investigating the use of benralizumab, an anti-IL-5Ra subunit monoclonal antibody. Nowak and colleagues reported the results of a phase two, randomized, double-blind, placebo-controlled study, where a single intravenous dose of 0.3 or 1 mg/kg of benralizumab or placebo was administered to patients who presented at the emergency department because of asthma exacerbation. The aim of this study was to evaluate whether a single dose of an anti IL-5Ra agent could reduce the future risk of exacerbation in patients who had recently experienced an acute episode of asthma. The authors concluded that benralizumab reduced asthma exacerbation rates by 49% (3.59 vs 1.82; *P* = 0.01) and the number of exacerbations requiring admission to hospital by 60% (1.62 vs 0.65; *P* = 0.02) in both groups (46). Interestingly, in a double-blind phase 2 study involving adult patients with uncontrolled asthma despite therapy with medium–high doses of ICS and long-acting β-agonists who had had between two and six exacerbations in the previous year, Castro et al. reported a relevant reduction in exacerbations in patients with a peripheral blood eosinophil count of at least 300/μL (70). On the other hand, improvements in lung function with anti-IL-5 or anti IL-5Ra monoclonal antibodies were less relevant (44, 45, 65, 70, 71). More recently, Bleecker and colleagues described the results of the SIROCCO study, a double-blind, parallel-group, placebo-controlled phase 3 clinical trial involving 12- to 75-year-old asthmatic patients with at least two exacerbations in the previous year despite optimal inhaled therapy. Patients were divided into two parallel arms. In the first, 400 patients received benralizumab 30 mg every 4 weeks and 398 patients every 8 weeks. In the second, 275 patients received 30 mg every 4 weeks and 267 patients every 8 weeks. The results confirmed the efficacy of benralizumab in severe eosinophilic asthma in terms of exacerbation and safety (72). It is noteworthy that similar results in terms of reduction in the frequency of exacerbations were obtained in the CALIMA study, which evaluated the efficacy of administering 30 mg of benralizumab every 4 or 8 weeks (47).

Changes in exacerbation rates have been shown to be favorable and encouraging overall, although improvements in FEV_1_ and in HRQOL did not reach statistical significance. Some studies showed improved pulmonary function (44, 64, 70, 71), whereas others did not (45, 46, 63, 65), and this variability in results was also described for HRQOL in other clinical studies (45, 46, 64, 65, 70, 71).

The fact that the patients enrolled in early trials did not show a significant reduction in the number of exacerbations could be due to two factors. First, in the case of baseline circulating eosinophilia, Castro et al. (69) found that the eosinophil cutoff value at baseline was higher than that described by Leckie and colleagues (60). Second, the exacerbation rate of the active population receiving the investigational product may have been different. In early trials, patients had mild-to-moderate asthma and a low number of asthma exacerbations in the previous year. In addition, the reduction appears less significant than that of active groups in later studies, which were characterized by higher baseline exacerbation rates and a more consistent reduction in the frequency of asthma flares (Table 1).

**Table 1:** Clinical trials on anti-interleukin 5 (IL-5) and IL-5Ra antagonists: main results and safety findings.

**Reference; study**	**Exacerbations**	**Other endpoints**	**Common adverse events**	**Serious adverse events**
**MEPOLIZUMAB**				
Chupp et al. (68); MUSCA study	Improvement in QoL	⇧ FEV_1_ (176 mL in mepolizumab group) ⇩ Exacerbations	Headache, nasopharyngitis, back pain, urticaria, arthralgia, arrhythmias, injection-site reactions	5% of patients in mepolizumab group has serious adverse events (asthma, systemic reactions)

Ortega et al. (44); MENSA study	⇩ Exacerbations (with intravenous medication, 47%; with subcutaneous administration, 53%)	⇧ FEV_1_ (100 mL intravenous administration, 98 mL subcutaneous administration)	Nasopharyngitis, upper respiratory tract infection, and headache	Incidence of 7% in intravenous group, 8% in subcutaneous, 14% in placebo

Bel et al. (64); SIRIUS study	⇩ Exacerbations (32%)	Improvement in Asthma Control Questionnaire 5 score	Headache, nasopharyngitis, injection-site reaction	Asthma exacerbations, pneumonia (both in placebo group)

Pavord et al. (45); DREAM study	⇩ Exacerbations (48% with 75-mg dose and 39% with 250-mg dose)	No change in FEV_1_ No change in ACQ scores	Headache, nasopharyngitis, infusion-related reaction	3 deaths (1 septic shock after acute pancreatitis, fatal asthma attack, suicide)

Nair et al. (73)	⇩ Exacerbations	⇩ Eosinophil count (in sputum and blood samples)	1 patient with shortness of breath (heart failure-related), 1 patient with aches and tiredness	No drug-related events recorded 1 death in placebo group

Haldar et al. (65)	⇩ Exacerbations (2.0 vs. 3.4 drug/placebo)	Improvement in AQLQ score ⇩ Eosinophil count (in sputum and blood samples) No change in FEV_1_	Facial flushing, rash, pruritus, erectile or ejaculatory dysfunction, fatigue	Severe acute asthma

Flood-Page et al. (63)	Reduction at higher doses of drug	No improvement in lung function or symptoms	Upper respiratory tract infection, asthma, headache, rhinitis, bronchitis, sinusitis, viral infection, injury, back pain, nausea, and pharyngitis	250 mg (hydrocephalus/cerebrovascular disorder, constipation, and gastrointestinal disturbance), 750 mg (asthma exacerbation)
**RESLIZUMAB**				
Castro et al. (69)	⇩ Exacerbations		Worsening of asthma symptoms, upper respiratory tract infections, nasopharyngitis	2 anaphylactic reactions

Castro et al. (71)	⇩ Exacerbations (less than other study)	⇩ Blood and sputum eosinophils, especially in patients with nasal polyposis Improvement in ACQ and lung function	Nasopharyngitis	Pneumonia, worsening of asthma
**BENRALIZUMAB**				
Bleecker et al. (72); SIROCCO study	⇩ Exacerbations – ⇩ 45% in Q4W – ⇩ 51% in Q8W	⇧ FEV_1_ – 106 mL Q4W – 159 mL Q8W Improved HRQoL	Nasopharyngitis, worsening of asthma	Allergic granulomatous angiitis, panic attack, paresthesia, injection-site erythema

FitzGerald et al. (47); CALIMA study	⇩ Exacerbations – ⇩ 36% in Q4W – ⇩ 28% in Q8W	⇧ FEV_1_ – 125 mL Q4W – 116 mL Q8W Improve of HRQoL	Nasopharyngitis, worsening of asthma	Urticaria, asthma, herpes zoster, chest pain

Nowak et al. (46)	⇩ Exacerbations	⇩ Blood eosinophils No improvement in lung function and HRQoL	Asthma, headache, dizziness, cough, pyrexia, bronchitis, anxiety, muscle spasms, and hyperhidrosis	Pyrexia, tachycardia, and anxiety

Castro et al. (70)	⇩ 40% Exacerbations in 100 mg group, but not in the 2 mg and the 20 mg groups	Dose–response findings ⇧ Lung function and HRQoL	Nasopharyngitis and injection-site reactions	100 mg: acute cholecystitis, herpes zoster, polyarteritis nodosa, and uterine leiomyoma 20 mg: erythema nodosum

Laviolette et al. (53)	⇩ Exacerbations		Nasopharyngitis, nausea One patient received an intravenous dose of 1 mg/kg: chills, headache, asthenia, nausea, dysgeusia, tremor, dizziness, hot flush, hyperhidrosis, and swelling with a decreased white blood cell count, decreased neutrophil count, and increase in C-reactive protein	Thyroid storm with hospitalization

### Safety

The safety data provided by clinical trials are generally reassuring, at least in the populations included (74). All IL-5 antagonists seem to be well tolerated. No deaths or severe reactions were reported for either intravenous or subcutaneous administration. The serious adverse events described in the trials with intravenous mepolizumab (hydrocephalus/cerebrovascular disorder, constipation, gastrointestinal disturbance, and asthma exacerbation) were not considered treatment-related by clinicians (63, 73). The SIRIUS study reported adverse events in 83% of patients receiving the drug subcutaneously and in 92% in the placebo group (see text footnote 1). The most common adverse events reported were headache, injection-site reactions, and nasopharyngitis, with similar frequencies in the active and placebo groups. Asthma exacerbations were reported in 3% of mepolizumab patients and in 12% of placebo patients (64). In one of the mepolizumab studies (MENSA), the frequency of adverse events was similar in all three groups: 84% in patients receiving intravenous mepolizumab, 78% in the subcutaneous group, and 83% in the placebo group. The reduction was significant if the events that were judged drug-related by clinicians (17% intravenous, 20% subcutaneous, and 16% placebo) are taken into account (44). Once again, the most common adverse events reported were nasopharyngitis, injection-site reactions, headache, and upper respiratory tract infections (44). In their trial on hypereosinophilic syndrome, Roufosse and colleagues reported the long-term safety results of 750 mg of mepolizumab administered every 9–12 weeks. The serious adverse events comprised four deaths, although only one was considered possibly drug related by clinicians. One patient died because of angioimmunoblastic T-cell lymphoma with cardiopulmonary failure (75). Three deaths were reported in the DREAM study: one case of septic shock secondary to pancreatitis (60-year-old woman), a fatal asthma attack (56-year-old man), and a case of suicide (54-year-old man) (45).

Adverse events with benralizumab in clinical trials were rare. Laviolette et al. reported a higher rate of adverse events in the benralizumab group than in the placebo group with both subcutaneous and intravenous administration (53). The most common adverse events were nasopharyngitis and headache in the intravenous group and nasopharyngitis and nausea in the subcutaneous group. One patient receiving 1 mg/kg of benralizumab intravenously experienced 15 adverse events [chills, headache, asthenia, nausea, dysgeusia, tremors, dizziness, hot flushes, hyperhidrosis, and swelling on day 0, with a decreased white blood cell count (2.3 × 10^3^/μL), decreased neutrophil count (1.1 × 10^3^/μL), and increased C-reactive protein level (1.61 mg/dL) measured on day 1 after dosing]. A patient with a prior history of hyperthyroidism, who was receiving 200 mg of benralizumab subcutaneously, experienced a thyroid storm 50 days after the first dose and 23 days after the last one. The patient was hospitalized for 8 days and subsequently completed the study. In this case, the physician considered the adverse event to be severe but not treatment related (53). Nowak and colleagues reported headache, dizziness, cough, pyrexia, bronchitis, anxiety, muscle spasms, and hyperhidrosis, with several serious events considered related to the study drug in four patients (pyrexia, tachycardia, and anxiety) (46). Castro reported adverse events in 72% of patients receiving benralizumab compared with 65% in the placebo group. The most common adverse events were nasopharyngitis and injection-site reactions (70). Safety may also be compromised by the onset of specific diseases secondary to the reduction in the eosinophil count and, therefore, potentially, the reduction in their protective effects. In this regard, no significant data have been reported or suspected in trials that explored these monoclonal antibodies. Additional data on safety *vis-à-vis* the reduction in the eosinophil count were added by Gleich et al., who claimed that depletion of these cells in both animal models and humans appears to have no harmful effects on health (76).

### Clinical Perspectives

Considering that almost all clinical trials performed with IL-5 and IL-5Ra antagonists showed favorable results in terms of efficacy and safety, new therapeutic perspectives can be hypothesized. Despite the encouraging results, the response to these drugs must be assessed in real life, although this is unlikely, since these drugs have only recently been marketed. Nevertheless, several recent studies have reported promising results in real life, for example, the MUSCA study in patients treated with mepolizumab (68). In daily clinical practice, clinicians will be required to identify the patients who will benefit most from these targeted therapies. The fact that IL-5 and IL-5Ra antagonists have been tested targeting not only severe asthma but also other diseases (some of which are associated, e.g., nasal polyposis) could pave the way for the discovery of biomarkers that enable the clinician to chose the right drug for the patient. In fact, the choice of one drug over another could, at least for the time being, be guided by comorbidities for which a drug has been tested and another not, choosing the one that covers both diseases. Although the evaluation of comorbidities could be an interesting starting point in patients with severe eosinophilic asthma, reliable biomarkers (i.e., periostin, eosinophils, IgE, and galectin-3) that predict the response to a targeted biological therapy are urgently needed.

## The Primary Role of Biomarkers

The development of new biological therapies to target not only IL-5 but also other T_H_2 cytokines including IL-4 and IL-13 (77, 78) and the incoming introduction of biosimilar drugs (79) raise the question of which therapy is best suited to a specific patient. The answer to this question may lie in the identification of biomarkers that predict therapeutic responses. An ideal biomarker should be easy to collect and evaluate, non-invasive, inexpensive, and sensitive (80). Many studies have proposed biomarkers for the management of asthmatic patients, including serum total IgE levels (IgEs) (81), exhaled nitric oxide (FeNO), blood and sputum eosinophil count (82, 83), and the possible role of galectin-3 obtained by bronchial biopsy in early studies (84) and now less invasively in serum (85) or serum periostin (86) as biomarkers of T_H_2-induced upper airway inflammation. Biomarkers are urgently needed to assign the most appropriate therapeutic strategy to a specific patient, according to the underlying asthmatic mechanism of inflammation. Consequently, therapy could switch from a “one size fits all” approach (the clinician prescribes a drug and, if this fails, a new prescription is made) to an approach based on “personalized medicine” or “precision medicine,” where a biomarker could help physicians to phenotype patients and choose the most appropriate therapy (87–90).

## Conclusion

For many years, eosinophilic inflammation has been considered a predominant mechanism in the development of asthma. IL-5 has been evaluated as a possible therapeutic target solely for its role in the development and action of eosinophils. Although initial trials results are not encouraging, it seems that choosing the optimal candidate, i.e., one with severe asthma and a high serum eosinophil count, could lead to a reduction in the frequency of exacerbations. The better results observed in patients with high eosinophil counts have led several authors to suggest >300–400/μL as a cutoff. Therefore, based on clinical trial results, it will be necessary to screen patients before prescription of anti-IL-5 and IL-5Ra drugs to choose the right patient for the right drug, i.e., one who is more likely to respond to a specific therapy. Eosinophils are currently the only suitable biomarker for these drugs, and every effort should be made to discover new biomarkers that enable more personalized and precise prescription. The role of biomarkers will become fundamental, leading clinicians to choose the best anti-IL-5 and IL-5Ra drugs for their patients.

## Author Contributions

DB and MF contributed equally to the conception and design of the study. DB, MF, FP, and GV drafted the manuscript. GC and GP approved the version to be published.

## Conflict of Interest Statement

GC has been a member of advisory boards and speaker at scientific meetings for GSK, Teva, Sanofi, Roche, Novartis, and Astra Zeneca. GP has been a consultant/speaker for ALK-Abelló, AstraZeneca, Lofarma, Novartis, and Stallergenes-Greer.

## Footnotes

^1^http://www.fda.gov/NewsEvents/Newsroom/PressAnnouncements/ucm471031.htm.

^2^http://www.fda.gov/newsevents/newsroom/pressannouncements/ucm491980.htm.

^3^http://www.ema.europa.eu/ema/index.jsp?curl=pages/medicines/human/medicines/003860/human_med_001933.jsp&mid=WC0b01ac058001d124.
